# Secondary attack rates from asymptomatic and symptomatic influenza virus shedders in hospitals: Results from the TransFLUas influenza transmission study

**DOI:** 10.1017/ice.2021.112

**Published:** 2022-03

**Authors:** Raphaël Tamò, Teja Turk, Jürg Böni, Roger D. Kouyos, Stefan Schmutz, Michael Huber, Cyril Shah, Heike A. Bischoff-Ferrari, Oliver Distler, Edouard Battegay, Pietro Giovanoli, Matthias Guckenberger, Malcolm Kohler, Rouven Müller, Heidi Petry, Frank Ruschitzka, Allison McGeer, Hugo Sax, Rainer Weber, Alexandra Trkola, Stefan P. Kuster

**Affiliations:** 1 Division of Infectious Diseases and Hospital Epidemiology, University Hospital and University of Zurich, Zurich, Switzerland; 2 Institute of Medical Virology, University of Zurich, Zurich, Switzerland; 3 Department of Geriatrics and Aging Research, University Hospital Zurich and University of Zurich, Zurich, Switzerland; 4 Department of Rheumatology, University Hospital Zurich, Zurich, Switzerland; 5 Department of Internal Medicine, University Hospital and University of Zurich, Zurich, Switzerland; 6 Department of Plastic Surgery and Hand Surgery, University Hospital and University of Zurich, Zurich, Switzerland; 7 Department of Radiation Oncology, University Hospital and University of Zurich, Zurich, Switzerland; 8 Pulmonary Division, University Hospital and University of Zurich, Zurich, Switzerland; 9 Department of Hematology, University Hospital and University of Zurich, Switzerland; 10 University Hospital Zurich, Zurich, Switzerland; 11 University Heart Center, University Hospital and University of Zurich, Zurich, Switzerland; 12 Department of Microbiology, Sinai Health System, Toronto Ontario, Canada

## Abstract

**Objective:**

Nosocomial transmission of influenza is a major concern for infection control. We aimed to dissect transmission dynamics of influenza, including asymptomatic transmission events, in acute care.

**Design:**

Prospective surveillance study during 2 influenza seasons.

**Setting:**

Tertiary-care hospital.

**Participants:**

Volunteer sample of inpatients on medical wards and healthcare workers (HCWs).

**Methods:**

Participants provided daily illness diaries and nasal swabs for influenza A and B detection and whole-genome sequencing for phylogenetic analyses. Contacts between study participants were tracked. Secondary influenza attack rates were calculated based on spatial and temporal proximity and phylogenetic evidence for transmission.

**Results:**

In total, 152 HCWs and 542 inpatients were included; 16 HCWs (10.5%) and 19 inpatients (3.5%) tested positive for influenza on 109 study days. Study participants had symptoms of disease on most of the days they tested positive for influenza (83.1% and 91.9% for HCWs and inpatients, respectively). Also, 11(15.5%) of 71 influenza-positive swabs among HCWs and 3 (7.9%) of 38 influenza-positive swabs among inpatients were collected on days without symptoms; 2 (12.5%) of 16 HCWs and 2 (10.5%) of 19 inpatients remained fully asymptomatic. The secondary attack rate was low: we recorded 1 transmission event over 159 contact days (0.6%) that originated from a symptomatic case. No transmission event occurred in 61 monitored days of contacts with asymptomatic influenza-positive individuals.

**Conclusions:**

Influenza in acute care is common, and individuals regularly shed influenza virus without harboring symptoms. Nevertheless, both symptomatic and asymptomatic transmission events proved rare. We suggest that healthcare-associated influenza prevention strategies that are based on preseason vaccination and barrier precautions for symptomatic individuals seem to be effective.

During influenza season, the prevalence of influenza virus in patients admitted to acute-care hospitals is high.^1–5^ Consequently, nosocomial transmission and outbreaks of influenza occur in acute care, and healthcare workers (HCWs) have been involved in transmission events.^6–9^ Even vaccinated individuals may be at risk of acquiring and transmitting the virus, especially because vaccination has been associated with oligo- or even asymptomatic illness.^10,11^

Up to one-third of all study participants with influenza virus infection do not develop symptoms. In addition, asymptomatic viral shedding usually precedes clinical illness by 1 day in individuals with symptomatic influenza.^12^ Although these facts are well acknowledged, viral shedding and transmission dynamics during asymptomatic influenza infection remain to be resolved.^13–15^

A precise understanding of transmission risks associated with asymptomatic individuals has fundamental implications for influenza prevention strategies in healthcare institutions. Therefore, we assessed in an acute-care hospital setting whether and to what extent influenza transmission from asymptomatic individuals occurred.

## Materials and methods

### Study setting, design, and participants

We conducted a prospective surveillance study at the University Hospital Zurich, a 900-bed tertiary-care center over 2 consecutive influenza winter seasons, 2015–16 and 2016–17.

The study methodology has been described in detail elsewhere.^16^ Active surveillance for influenza infection was performed in all consenting inpatients hospitalized and all consenting HCWs working on the same wards. The following HCWs were included: nursing staff, medical staff, corporate hospitality staff with direct patient contact, and physiotherapists.

We determined the start of the influenza winter season when the national threshold for an influenza epidemic according to the Swiss Sentinella Surveillance System was reached.^17^ The sustained drop of national influenza cases below this threshold for 2 consecutive weeks defined the end of influenza season.

Inpatients were recruited upon admission to the study wards. From recruitment to discharge from the study ward, a study nurse collected daily flocked mid-turbinate nasal swabs for influenza virus detection by polymerase chain reaction (PCR). The nurse also conducted a short interview using a questionnaire asking for symptoms of influenza infection, contact with persons (HCW or visitors) suffering from symptoms of influenza infection, and prior receipt of influenza vaccine.^18^ Daily measurements of body temperature were extracted from inpatient electronic medical records during their hospital stay. Patients were asked to self-collect daily nasal swabs, questionnaires, and body temperature for 2 more days after discharge from the study ward.

HCWs were enrolled prior to the influenza season. During the entire influenza season, HCWs proceeded with daily self-collection of flocked midturbinate nasal swabs for influenza PCR. They also completed illness diaries with symptoms of influenza infection, contact with coworkers, inpatients or family member suffering from symptoms of influenza infection, and study patients cared for.

To avoid bias, the results of the flocked mid-turbinate nasal swabs from inpatients and HCWs were not made available to study participants and treating physicians until the end of the study. Testing for and treatment of influenza infection in inpatients according to clinical suspicion was the responsibility of the treating physicians, and infection prevention and control precautions were guided by the study-independent local infection control team.

### Contact tracing

Contacts among HCWs, among inpatients, and between HCWs and inpatients were tracked through work schedules, bed occupation plans, and contacts registered in the electronic medical records, respectively. The contacts in the electronic patient record system were provided by HCWs on a specific study tab, and routine electronic activity was also analyzed. A contact was defined as eye contact with physical contact (eg, handshake) or a distance between faces of study participants of <1 m if there was no physical contact between study participants. Once the contacts were traced, the influenza transmission chains were reconstructed.

### Laboratory analyses

*Influenza detection and whole-genome sequencing.* Samples were analyzed in the accredited diagnostics laboratory of the Institute of Medical Virology (University of Zurich, Switzerland). Virus transport medium from flocked midturbinate nasal swab samples was homogenized, divided into aliquots, and stored at −20°C until use. Using a multiplex real-time PCR influenza A (pan A), influenza B (pan B) and amplification inhibition could be detected and discriminated.

To allow for tracing of transmission chains of influenza virus, the near full-length genome was amplified directly from the specimens that tested positive for influenza virus RNA, and sequencing was performed based on next-generation sequencing.^19,20^

*Phylogenetic analyses of transmission events.* Likely transmission events from symptomatic or asymptomatic patients were identified through epidemiological analyses. Such likely transmission events were then further corroborated or refuted based on the phylogenetic analysis (see Supplementary Materials online for details). Near full-length genome sequences (Supplementary Table S1 online) were used for characterization of possible transmission events independently. Briefly, study sequences were pooled with viral sequences from the community. Using BLAST, the most similar foreign background sequences were identified.^21^ Subsequently, phylogenetic trees were built using PhyML alongside 1,000 bootstrap trees.^22^ Potential transmission clusters were defined as subtrees with maximum pairwise (GTR+Γ substitution model-derived) genetic distance below a specified threshold and with sufficiently high bootstrap support (≥90%). To the best of our knowledge, there are no consensus thresholds to detect direct transmission of influenza virus. Based on participants with multiple sequences (Supplementary Table S2 online), we considered a range of thresholds (Supplementary Fig. S2 online) and deductively evaluated their plausibility with respect to prevalence of multiple infections, contact data, and duration of infection and viral shedding. Thus, we were able to determine reasonable thresholds to be used for the main analysis.

### Statistical analysis

In our primary analysis, we described and calculated the secondary attack rate of asymptomatic or presymptomatic influenza. Secondary analyses included the secondary attack rates of symptomatic influenza infection and the proportions of asymptomatic and symptomatic participants with influenza among inpatients and HCWs, risk factors for influenza infection, and the association of individual influenza symptoms with viral shedding quantified by cycle threshold (C_t_) values from RT-PCR.

Categorical data were tested for differences using the Fisher exact test, whereas continuous variables were tested using Wilcoxon rank-sum tests or the Student *t* test, as appropriate. Multivariable regression analysis was used to assess independent risk factors or predictors for influenza infection and semiquantitative influenza shedding, respectively. Clustering was accounted for, as appropriate, if multiple measurements from the same individual were included. We considered the following potential risk factors for influenza infection: participant characteristics, study season (see Supplementary Table S4 online) and for analysis of semiquantitative influenza shedding: cough, sore throat, fever ≥38.0°C, nasal congestion, weakness, headache, loss of appetite or myalgia. In univariable analyses, variables with *P* values < .10 were considered for inclusion in multivariable models based on clinical judgment.^23^

### Ethical considerations

The study protocol was approved by the cantonal ethics committee of the Canton of Zurich (KEK-ZH-Nr.: 2015-0228). Informed consent of all participants was obtained before enrollment, and withdrawal from the study was possible at any time.

## Results

### Recruitment and adherence to the study protocol

In total, 152 HCWs and 542 inpatients from 8 wards in the 2015–16 and 2016–17 influenza seasons were included, accounting for 40.9% of all eligible healthcare workers and 29.8% of all eligible inpatients.

### Healthcare worker and inpatient characteristics

Supplementary Table S4 (online) depicts characteristics of study participants in the 2 influenza seasons. The median duration of study participation was longer in HCWs in the 2015–16 influenza season (median, 96 days; range, 71–96) than in the 2016–17 influenza season (median, 66 days; range, 25–67; *P* < .001) due to a longer duration of influenza circulation in Switzerland during the former season.

### Influenza cases and symptoms

Overall, 35 participants (5.0%), 16 HCWs (10.5%), and 19 inpatients (3.5%) were diagnosed with influenza, and 31 (88.6%) of these were infected with influenza A: 13 with influenza A/H1N1 in the 2015–16 season and 1 with influenza A/H1N1 and 17 influenza A/H3N2 in the 2016–17 season. All 4 influenza B cases were detected in the 2015–16 influenza season. Of 19 influenza infections in inpatients, 8 (42.1%) were healthcare-associated, and 2 (25.0%) of these 8 were acquired in other institutions. In total, 1,241 swabs were collected in these 35 study participants. Of these, 109 (8.8%) swabs tested positive. The number of positive swabs per individual ranged from 1 to 13.

**Fig. 1.**
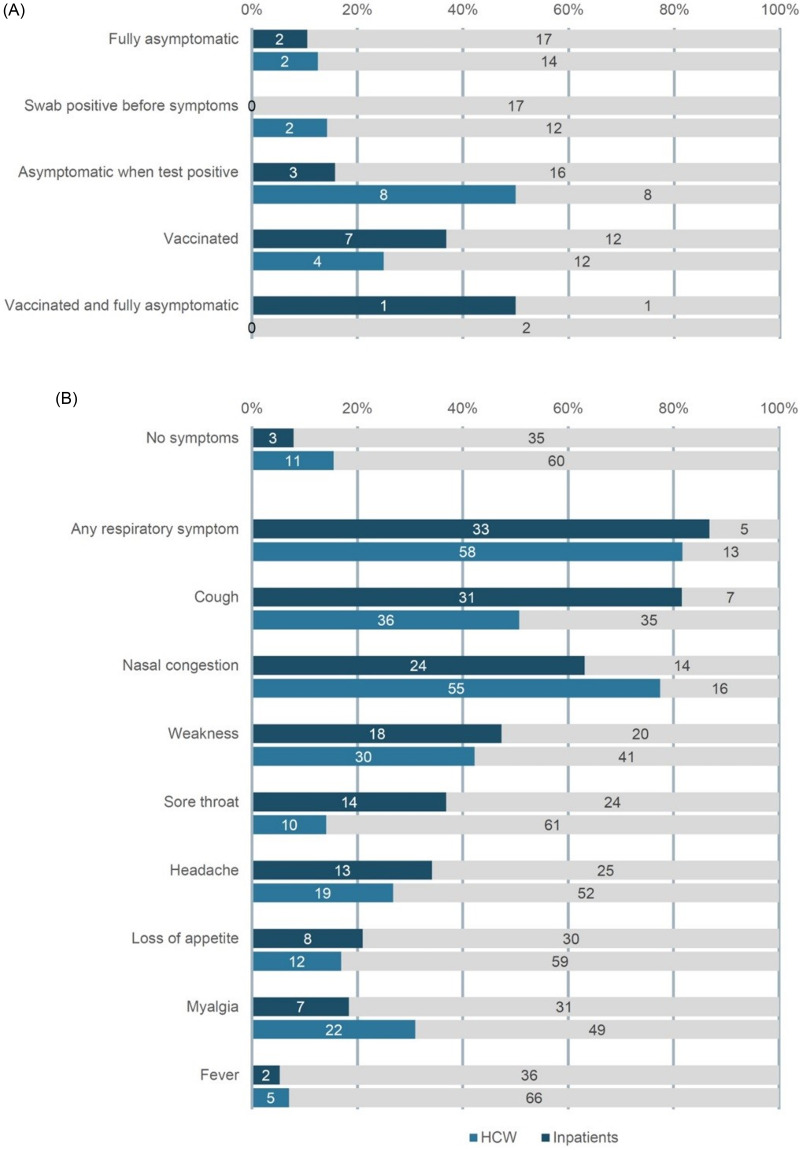
Signs and symptoms of 16 healthcare workers and 19 patients (panel A) and in relation to days with positive test results (panel B) diagnosed with symptomatic or asymptomatic influenza infection during the 2015–16 and the 2016–17 influenza seasons, University Hospital Zurich.

The clinical presentation of participants with influenza infection is depicted in Fig. 1. Moreover, 2 (12.5%) of 16 HCWs and 2 (10.5%) of 19 inpatients remained fully asymptomatic (Fig. 1, panel A). Receipt of influenza vaccine was not associated with asymptomatic shedding of influenza virus: 3 (27.3%) of 11 participants with asymptomatic shedding and 8 (33.3%) of 24 participants without asymptomatic shedding had received vaccine (*P* = 1.00). Most participants (83.1% of HCWs and 92.1% of inpatients) had signs and symptoms of influenza infection when their tests were positive, and these were mostly respiratory symptoms (Fig. 1, panel B). Notably, 11 (15.5%) of 71 influenza-positive swabs in 8 different HCWs and 3 (7.9%) of 38 influenza-positive swabs in 3 different inpatients were collected on days without symptoms.

None of the participant characteristics listed in Supplementary Table S4 (online) were associated with influenza infection. Particularly, the risk of influenza infection was not affected by receipt of vaccine: 24 (4.9%) of 486 unvaccinated participants and 11 (5.3%) of 208 vaccinated participants were diagnosed with influenza (*P* = .85). We detected no independent association between influenza risk and vaccination status or influenza season.

In multivariable analyses, C_t_ values were lower in study participants shedding influenza A/H1N1 with fever (mean C_t_ value, 29.3; SD, 0.05) than in those without (mean C_t_ value, 34.0; SD, 5.7; *P* ≤ .001).

### Contacts between study participants with influenza, influenza clusters and transmission events

Overall, we identified 172 contacts between symptomatic and other study participants and 65 contacts between asymptomatic participants with influenza shedding and other study participants (Table 1). For secondary contacts, at least 1 swab was collected in the 4 days following the contact for 159 (92.4%) of 172 symptomatic index cases and 61 (93.8%) of 65 asymptomatic index cases. HCWs reported that they wore masks during work on 18 (81.8%) of 22 days with symptomatic influenza shedding. Patients with influenza shedding were under droplet precautions during 1 (33.3%) of 3 contacts when asymptomatic and during 17 (51.5%) of 33 contacts when suffering signs and symptoms of influenza infection.

**Table 1.** Contacts of Healthcare Workers and Inpatients With Influenza Infection With Other Study Participants During the 2015–16 and the 2016–17 Influenza Seasons, University Hospital Zurich

Characteristic	Influenza-Positive Study Participants	
	HCW (N = 16)	Inpatients (n = 19)
No. of days present in hospital with influenza shedding	30	38
No. of days without symptoms, n/N (%)	8/30 (26.7)	3/38 (7.9)
No. of susceptible HCWs in contact with influenza positive index person	152	36
No. of HCWs in contact on days with asymptomatic shedding, n/N (%)	44/152 (28.9)	3/36 (8.3)
No. of susceptible patients in contact with influenza positive index person	49	0
No. of patients in contact on days with asymptomatic shedding, n/N (%)	18/49 (36.7)	0 (0)

Note. HCW, healthcare worker.

**Fig. 2.**
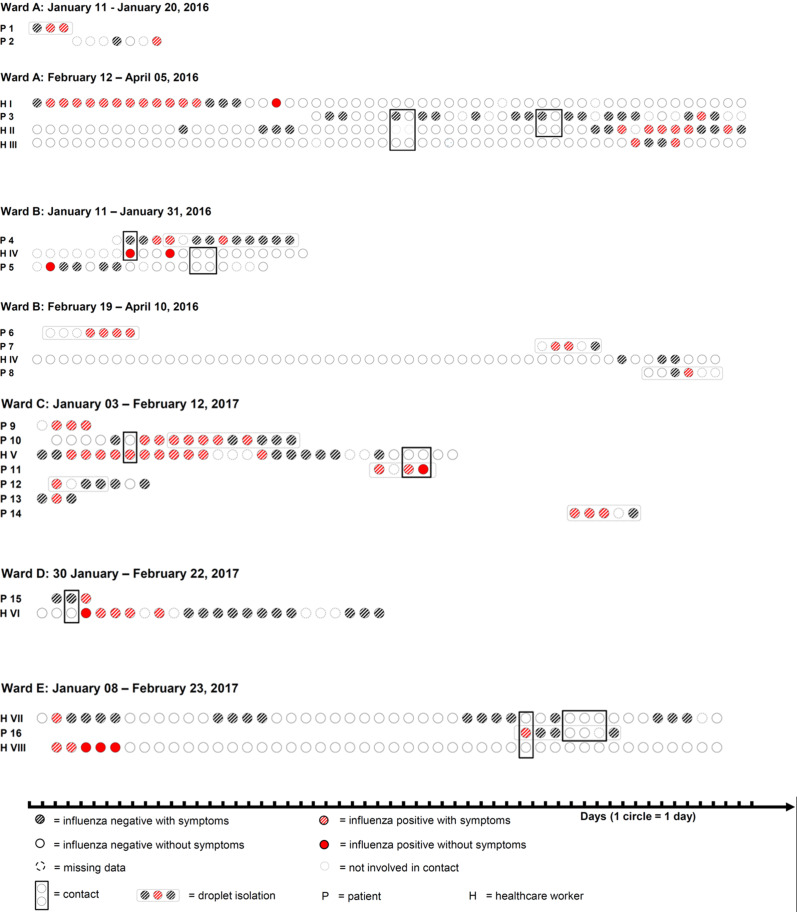
Clusters of influenza cases in healthcare workers and patients according to temporal and spatial proximity, University Hospital Zurich, during the 2015–16 and 2016–17 influenza seasons.

Analyses based on spatial and temporal proximity of HCWs and inpatients revealed 7 clusters of potential influenza transmission events among HCWs, among inpatients or between HCWs and inpatients (Fig. 2). All clusters consisted of patients infected with influenza A. One cluster suggested a possible transmission from an asymptomatic HCW to an inpatient (Fig. 2, ward B, from HCW H-IV to patient P4). Another cluster suggested transmission from a symptomatic patient to an HCW (Fig. 2, ward D, from patient P15 to HCW H-VI).

Phylogenetic analyses, however, did not support the asymptomatic transmission event on ward B (Supplementary Fig. S5 online). More precisely, in only 4 (0.4%) of 1,000 bootstrap trees but not in the most likely phylogenetic tree (Supplementary Fig. S5 online), the samples of P4 and H- IV formed a monophyletic group (case 1a in Supplementary Fig. S1 online). In addition, the minimum genetic distance between their sequences was 0.006329, which was considerably larger than the largest reasonable upper bound distance (0.0011) of a true transmission cluster (Supplementary Fig. S2 online and Supplementary Fig. S3 online). With the genetic distance threshold of 0.00045, 2 of the previously identified potential transmission pairs could be corroborated from P15 to H-VI on ward D and between the patients P9 and P10 on ward C. The phylogenetic evidence was stronger in case of the first potential transmission pair (P15 to H-VI) because the MRCA clade of H-VI also contained the sample of P15 (case 2b from Supplementary Fig. S1 online, occurring in 74.4% of the bootstrap trees), but this was not the case for the second pair (case 3b from Supplementary Fig. S1 online, similar in 51.6% of the trees). The genetic distances and bootstrap support values were similar in both cases (0.00041 and 99.9%, respectively). These findings were also consistent with the epidemiological tracing data (Fig. 2). Namely, while P15 and H-VI had a direct contact, only weak spatial proximity could be detected between P9 and P10 because both patients were placed in adjacent rooms without direct epidemiologic contact.

Therefore, based on temporal and spatial proximity and phylogenetic analyses and including only secondary contacts with at least 1 swab collected in the 4 days following the contact, the secondary transmission rate was 1 single transmission event in 159 days with contact (0.6%; 95% confidence interval, 0.02%–3.5%) for infections originating from symptomatic cases and no transmission event in 61 days (0%; 1-sided, 97.5% confidence interval, 0.0%–5.9%) with contact for those originating from asymptomatic individuals.

## Discussion

In a large, prospective surveillance study involving 152 HCWs and 542 hospitalized patients and the collection of >10,500 mid-turbinate nasal swabs and 11,000 illness diaries in the 2015–16 and 2016–17 influenza seasons, we were unable to detect a single case of asymptomatic transmission of influenza virus that was evident from spatial and temporal proximity and supported by phylogenetic analysis. Our results indicate that even though asymptomatic shedding does occur and transmission in the absence of respiratory symptoms has been hypothesized,^12,15,24–27^ transmission from asymptomatic shedders in a real-life acute-care setting seems to be rare if not completely absent. On the other hand, influenza infections, both symptomatic and asymptomatic, are a frequent event in the acute-care hospital setting, especially in HCWs. Thus, several clusters of infection were detected, but only 1 transmission event that was weakly supported by phylogenetic analyses was identified between a symptomatic inpatient that transmitted influenza virus to an unvaccinated HCW while not being under isolation precautions.

We are not aware of any other prospective study that has rigorously assessed transmission pathways of influenza infection in asymptomatic individuals in a real-life acute-care setting.^15,27–29^ It has been repeatedly claimed that contacts are at risk for influenza infection when being exposed to someone with asymptomatic viral shedding. Our results indicates that such risk is probably remote. Our study was conducted over 2 influenza seasons with 2 different dominant circulating strains (A/H1N1 in 2015–16, A/H3N2 in 2016–17) in Switzerland, and there was no antigenic mismatch between circulating strains and vaccine strains in both influenza seasons, but vaccine effectiveness was nevertheless limited.^30,31^ Therefore, there is limited risk that strain-specific effects or a mismatch between influenza vaccine and circulating strains would play a major role.

Our study has several limitations. It was a prospective, open-label surveillance study in a single, tertiary-care institution in a high-income country. Our results may not be generalizable to other geographic areas or different acute or long-term care institutions. Nevertheless, the infection control precautions in place at the study site (ie, hand hygiene policy and self-masking in the presence of respiratory symptoms) and sick leave during influenza-illness symptoms are comparable to those of other institutions. Sample size issues due to limited participation rates in both patients and HCWs may have prevented us from detecting asymptomatic transmission events; the total number of participants with influenza was low in our study. In 6 HCWs who worked when being asymptomatic on a day when they tested positive, and 3 additional asymptomatic inpatients, we recorded contacts with 61 other study participants that provided at least 1 swab within the 4 days following the contact. Even if we assumed that there was only 1 contact with each other study participant, this would result in a power of 79% to detect a transmission with a secondary attack rate of 2.5% and a 96% power to detect a transmission with a secondary attack rate of 5%. Nevertheless, the total number of asymptomatic shedding days was low, and we cannot rule out that individual participant characteristics may have contributed to a lower attack rate. A major limitation of our study was that we were unable to track contacts between study participants down to the resolution of individual face-to-face contacts. However, our estimate of only 1 face-to-face contact on days with asymptomatic shedding is rather conservative. However, some HCWs may only have had transient contacts with each other during a single shift.

Apart from asymptomatic transmission events, there was also no transmission from a symptomatic HCW to an inpatient. As shown in a previous analysis, a considerable fraction of 67.9% of all participating HCWs worked with symptoms of influenza infection on 8.8% of study days,^32^ but symptomatic HCWs with proven influenza infection wore masks during work on 18 of 22 days with symptoms. A Hawthorne effect may have contributed to good adherence with mask use during these days and may be less pronounced in other individuals. Nevertheless, the study reflects a real-life situation with gaps in vaccination rates and mask use among HCWs and delays in the initiation of droplet precautions, and our findings suggest that these measures are sufficient to prevent transmissions from symptomatic individuals.

In the past, the pathways of spread through individual communities have been revealed through phylogenetic analysis,^33^ which is capable of disclosing possible transmissions even in the absence of a documented contact. Only 1 pair of 2 patients without evident direct contact suggested such an event, indicating that the number of transmissions through individuals that did not participate in the study may have been low.

In conclusion, our results indicate that even though asymptomatic influenza shedding does occur in HCW and inpatients in acute-care hospitals with multifaceted influenza prevention polices, transmission events originating from these individuals seem to be rare if not inexistent.

## Supplementary material

For supplementary material accompanying this paper visit https://doi.org/10.1017/ice.2021.112. click here to view supplementary material
