# Supplementary material for: Secondary attack rates from asymptomatic and symptomatic influenza virus shedders in hospitals: Results from the TransFLUas influenza transmission study
Source: Infect Control Hosp Epidemiol. 2021 May 6;43(3):312–8. doi: 10.1017/ice.2021.112 (PMC8961411; doi:10.1017/ice.2021.112)

**Supplemental materials**

**Exclusion criteria for study participants**

Subjects under the age of 18, those with impaired judgement due to language problems, psychological disorders or dementia, individuals with known or suspected non-compliance or non-availability for follow-up during the study period were excluded. Specific exclusion criteria for HCW only were employment of less than 50%, planning to spend more than two consecutive weeks outside of Switzerland during the winter study period (1^st^ November to 31^st^ April) or planning to take leave from work for more than two consecutive weeks during the winter study period (e.g. maternity or medical leave).

**Sample size considerations**

Assuming an average duration of influenza season of 15 weeks (*1*), a proportion of HCW with asymptomatic or presymptomatic influenza of 17%(*2*), a proportion of inpatients with asymptomatic or presymptomatic influenza of 2.5% (*3, 4*), a drop-out rate of 10%, and a secondary attack rate of asymptomatic or presymptomatic influenza of 5% (*3, 5-8*), we calculated the number of contacts required to detect one single influenza transmission from an asymptomatic or presymptomatic index case with a power of 80%, to 33, and a corresponding number of 840 inpatients and 120 HCW providing a total of 10,080 samples needed. Corresponding numbers for a secondary attack rate of 3% were 54 contacts, 1,260 inpatients, 180 HCW, and 15,120 swabs.

**Viral sequences**

Whole influenza genome background sequences were retrieved from the NCBI Influenza Virus Resource sequence database using the Full Genome Sets Interface (*9*). We restricted our search to complete genomes only with human host and collection date between November 2014 and April 2018. In addition to the foreign background sequences, we also included nearly full length viral sequences from the Zurich region collected during the same two influenza seasons as in our study. Since the regional sequences are spatially closer than the foreign sequences, they should in principle improve the separation of the sequences from the study, especially in case of community introduced infections in the hospital.

**Phylogenetic tree construction**

Since all the available study sequences were either entirely influenza A virus (IAV) subtype H1N1 or entirely of subtype H3N2, we restricted the background sequences to the same subtypes. The workflow described in the following paragraphs was applied separately to both subtypes.

First, the segments from the same sample were concatenated to form near full-length viral sequences. Next, using Nucleotide BLAST 2.2.31+ with 10 maximum target sequences, maximum E-value 0.05 and percentage identity threshold of 90%, the background sequences most similar to the community and study samples were identified (*10*).

Afterwards, five sequences were randomly selected among the foreign background sequences of the complementary subtype (i.e. H3N2 for H1N1, and vice versa) to constitute the outgroup. Outgroup, blasted background, majority consensus community and study sequences (Supplemental Table S1) were pooled together and aligned with MUSCLE (*11*).

Finally, a phylogenetic tree was built using PhyML 20120412 with GTR+Γ nucleotide substitution model, 8 relative substitution rate categories and 1000 bootstrap trees (*12*). For the remaining input parameters, the default values were used. The resulting trees were further processed and analyzed in R version 3.5.2, employing R-packages ape and seqinr, and custom written scripts (*13, 14*).

**Transmission clusters extraction**

Each phylogenetic tree was rooted using the pre-defined outgroup as described above and the most recent common ancestor (MRCA) clade of the ingroup was extracted (Supplemental Figure S5). For a given maximum genetic distance threshold $d_{max}$ and a bootstrap support value $B$ we defined a cluster as a clade with

1. bootstrap support value greater or equal to $B$ (namely the same set of sequences formed a monophyletic group in at least $B$ out of 1000 bootstrap trees); and
2. genetic distance smaller or equal than $d_{max}$ between any two of its tips.

This kind of cluster definition has been utilized elsewhere. Notably, it was used for the implementation of ClusterPicker and applied to different viruses, including human immunodeficiency, hepatitis C and influenza virus.^7^

We declared such a phylogenetic cluster as a potential transmission cluster if it contained sequences of either a) at least two different study participants or b) one study participant and at least one non-study sample. Furthermore, study participants with multiple sequences were interpreted as being co-infected (under the considered cluster definition) if the MRCA clade of their samples did not fulfill the criteria for being a cluster given the thresholds under consideration. In other words, if viral sequences from the same person could be split into at least two groups that were genetically too distinguishable to belong to the same transmission cluster, this person was considered to be infected with different strains.

Supplemental Figure S1 visualizes why identification of the transmission cluster based on the MRCA composition alone is not sufficient, especially when the sampling is sparse (as it is arguably the case for influenza): while the samples of two persons might form a monophyletic group (Supplemental Figure S1, panels 1.b—3.b), it could be that some of the intermediate hosts/transmitters’ viral sequences were missed (Supplemental Figure S1, panels 1.a—3.a) and hence falsely give the impression of a transmission cluster. While the bootstrap support value threshold ensures sufficient certainty of the clade composition, the maximum pairwise genetic distance can be used to identify sequences which are genetically too different to be a result of a recent branching event, i.e. transmission. Intuitively, viral evolution of the transmitted virus should be in line with the within host viral evolution of the transmitter over a similar time range. Thus, if the smallest genetic distance between sequences from two different persons is much larger than the genetic distance observed within a single person, this is a strong indication against direct transmission. Additionally, it is important to notice that a within host viral evolution could be a result of multiple infection events,^8,9^ hence overestimating the plausible range of genetic distance within one patient. To summarize, large genetic distances and presence of background sequences in the MRCA clades strongly support the absence of a direct transmission event.

Using deductive reasoning, we considered a range of thresholds and compared the number of transmission clusters with the number of co-infected individuals claimed by each premise (Supplemental Figure S2). The absence of any set of thresholds yielding 0 transmission events and 0 coinfections (“0/0” field) in case of H3N2 indicated either at least one transmission cluster or at least one co-infected study participant regardless of the thresholds.

With bootstrap support value threshold of 90% and maximum pairwise genetic distance 0.0011 our method identifies three clades as potential transmission clusters – A, B and C (Supplemental Figure S3). Next, we investigated three possible premises (assuming $B$=900) and their plausibility with respect to the frequency of co-infections, and duration of infection and viral shedding:

- *Premise a (with* $d_{max}$*=0.000400): none of the clades A, B or C forms a transmission cluster*

Under this assumption, even the earlier three samples of H VI do not form any transmission cluster, suggesting a co-infection with at least two strains (Supplemental Figure S3). Additionally, except for H III, H V, H XIII and P 11, none of the participants with multiple samples would form a transmission cluster (Supplemental Table S2). This thesis thus claims 7 coinfections (5 due to too high genetic distance) among 12 influenza cases with multiple available samples. Even though there is not consensus about the frequency of such infections,^8,10^ 58.3% (7 out of 12) in our case would even exceed the debatably highest observed frequency of 50%.^10,11^ This premise hence arguably imposes a too strict threshold on genetic distance.

- *Premise b (with* $d_{max}$*=0.000411): clade A forms a transmission cluster, clades B and C are not transmission clusters*

Since the only sample of participant P 15 was nested within the MRCA of H VI’s samples in 744 of 1000 bootstrap trees (topology 2.b from Supplemental Figure S1, Supplemental Table S1), this is indicative of clade A forming a transmission cluster. Under these assumptions, however, three H1N1-infected individuals and three H3N2-infected participants would still be considered co-infected. Assuming that mixed infections could be rare,^8,12^ with 50% of co-infections this threshold for the genetic distance is still arguably too conservative.

- *Premise c (with* $d_{max}$*=0.001050): clades A and B represent transmission clusters, clade C is not a transmission cluster*

This proposition is consistent with absence of any co-infections and two possible transmission clusters. It is noteworthy that the P 10’s sample was nested within P 9’s samples in 484 out of 1000 bootstrap replicates (case 2.b, Supplemental Figure S1), hence the evidence of clade being a real transmission cluster is not strong. On the other hand, if the assumptions were relaxed further, the clade C would have been identified as a transmission cluster. Due to absence of direct contact between H XIII and P 11, this is highly unlikely. In addition, the H XIII’s swabs were tested negative from the fourth day onwards after being positive for the first time and the HCW self-reported wearing a mask at work throughout the duration of symptoms. Since the time lag between the positive samples from P 11 and H XIII is rather long (10 days), this imposes additional argument for why this transmission is rather implausible.

In summary, our model thus supports that H VI and P 15 are a transmission pair. We furthermore identified another possible candidate transmission pair for which the evidence is weaker (the proportion of MRCAs with nested samples was only 48.4% compared to 74.4% in case of H VI and P 15). In summary, genetic distance greater than 0.0011 suggests two or more infection events and is hence supportive for the absence of a direct transmission. The genetic distances below this threshold should be taken with care as proclaiming all the transmission clusters as real transmission clusters could result in falsely identified transmission events. Note however, that the transmission pair (H VI, P 15) claimed in this study is also supported by epidemiological evidence – in particular, contact tracing – and hence the phylogenetic evidence is only used to corroborate or refute the epidemiologically inferred pairs.

**References**

1. Paget J, Marquet R, Meijer A, van der Velden K. Influenza activity in Europe during eight seasons (1999-2007): an evaluation of the indicators used to measure activity and an assessment of the timing, length and course of peak activity (spread) across Europe. BMC Infect Dis. 2007;7:141.

2. Kuster SP, Shah PS, Coleman BL, Lam PP, Tong A, Wormsbecker A, et al. Incidence of influenza in healthy adults and healthcare workers: a systematic review and meta-analysis. PLoS One. 2011;6(10):e26239.

3. Carrat F, Vergu E, Ferguson NM, Lemaitre M, Cauchemez S, Leach S, et al. Time lines of infection and disease in human influenza: a review of volunteer challenge studies. Am J Epidemiol. 2008;167(7):775-85.

4. Patrozou E, Mermel LA. Does influenza transmission occur from asymptomatic infection or prior to symptom onset? Public Health Rep. 2009;124(2):193-6.

5. Iten A, Manuel S, Pagani L, Thomas Y, Camus V, Kaiser L, et al. Epidemic of Seasonal Influenza in an Internal Medicine Service. Abstract K-956. 52nd Interscience Conference on Antimicrobial Agents and Chemotherapy, San Francisco, USA, 2012.

6. Lucker LM, Kherad O, Iten A, Wagner N, Descombes M, Camus V, et al. Clinical features and outcome of hospitalised adults and children with the 2009 influenza A H1N1 infection at Geneva's University Hospital. Swiss Med Wkly. 2011;141:w13177.

7. Pagani L, Sauvan V, Iten A, Kaiser L, Harbarth S, Pittet D. Nosocomial Outbreak of Seasonal Influenza in a Geriatric Hospital. Abstract K-954. 52nd Interscience Conference on Antimicrobial Agents and Chemotherapy, San Francisco, USA, 2012.

8. Siegrist CA, van Delden C, Bel M, Combescure C, Delhumeau C, Cavassini M, et al. Higher memory responses in HIV-infected and kidney transplanted patients than in healthy subjects following priming with the pandemic vaccine. PLoS One. 2012;7(7):e40428.

9. Influenza Virus Genome Set: National Center for Biotechnology Information; [Available from: <https://www.ncbi.nlm.nih.gov/genomes/FLU/Database/nph-select.cgi?go=genomeset>.

10. Basic Local Alignment Search Tool: National Center for Biotechnology Information; [Available from: <https://blast.ncbi.nlm.nih.gov/Blast.cgi>.

11. Edgar RC. MUSCLE: multiple sequence alignment with high accuracy and high throughput. Nucleic Acids Research. 2004;32(5):1792-7.

12. Guindon S, Dufayard J-F, Lefort V, Anisimova M, Hordijk W, Gascuel O. New Algorithms and Methods to Estimate Maximum-Likelihood Phylogenies: Assessing the Performance of PhyML 3.0. Systematic Biology. 2010;59(3):307-21.

13. Paradis E, Claude J, Strimmer K. APE: Analyses of Phylogenetics and Evolution in R language. Bioinformatics. 2004;20(2):289-90.

14. Charif D, Lobry JR. SeqinR 1.0-2: a contributed package to the R project for statistical computing devoted to biological sequences retrieval and analysis. Structural approaches to sequence evolution: Springer; 2007. p. 207-32.

**Supplemental Table S1** Overview of the near full-length sequences used in the phylogenetic analysis workflow.

|  | **Influenza A/H1N1** | | | **Influenza A/H3N2** | | |
| --- | --- | --- | --- | --- | --- | --- |
|  | 2015/16 | 2016/17 | **Overall** | 2015/16 | 2016/17 | **Overall** |
| Study sequences, *n* | 23 | 1 | **24** | 0 | 31 | **31** |
| Study participants with at least one sequence, *n* | 8 | 1 | **9** | 0 | 12 | **12** |
| Study participants with multiple sequences, *n* | 5 | 0 | **5** | 0 | 7 | **7** |
| Community sequences, *n* | 16 | 0 | **16** | 5 | 16 | **21** |
| Background sequences, *n* | - | - | **2990** | - | - | **6718** |
| Blasted background sequences, *n* | - | - | **62** | - | - | **66** |
| Outgroup sequences, *n* | - | - | **5** | - | - | **5** |

**Supplemental Table S2.** Summary of the most recent common ancestor (MRCA) clades of participants with multiple viral sequences. The bootstrap support value indicates the support value of MRCA clade, whereas the percentage phylogenetic corresponds to the percentage of the bootstrap trees in which the MRCA clade was monophyletic, namely it contained exclusively the participant’s samples. The genetic distance – MRCA summarizes the maximum pairwise genetic distance between any two tips of the MRCA clade, while the genetic distance – participant’s samples shows the maximum pairwise genetic distance between any two sample of the same participant.

| Study participant | Influenza A virus serotype | Bootstrap support value | Percentage monophyletic [%] | Genetic distance – MRCA [10^-6^] | Genetic distance – participant’s samples [10^-6^] |
| --- | --- | --- | --- | --- | --- |
| H III | H1N1 | 1000 | 100 | 308.32 | 308.32 |
| H I | H1N1 | 994 | 99.4 | 463.95 | 463.95 |
| H XI | H1N1 | 993 | 99.3 | 619.32 | 619.32 |
| P 1 | H1N1 | 999 | 99.9 | 465.66 | 465.66 |
| P 4 | H1N1 | 1000 | 100 | 310.54 | 310.54 |
| H V | H3N2 | 1000 | 100 | 0.10 | 0.10 |
| H XIII | H3N2 | 943 | 94.3 | 138.57 | 138.57 |
| P 9 | H3N2 | 516 | 51.6 | 0.06 | 0.06 |
| P 10 | H3N2 | 861 | 86.1 | 138.62 | 138.62 |
| H VI | H3N2 | 999 | 25.6 | 410.74 | 410.74 |
| P 11 | H3N2 | 985 | 98.5 | 136.81 | 136.81 |
| P 14 | H3N2 | 1000 | 100 | 410.35 | 410.35 |

**Supplemental Figure S1.** Elements of transmission cluster definition. Panels 1—3 show some possible topologies for MRCA clades of samples from 2 different persons A (blue) and B (rosa). Triangles denote the subtrees, containing only samples from the corresponding persons or exclusively samples not belonging to A or B (marked with X, in gray). The configurations **b** show the topologies in case of incomplete sampling, namely, when the samples belonging to the X subtrees from **a** are not sampled. The uncertainty of the composition of MRCA clades is reflected in the bootstrap support value. Panel 4 summarizes the inversely proportional relationship between the probability that a candidate pair is a real transmission pair depending on the genetic similarity between their viral sequences – the larger the pairwise genetic distance the less likely the true direct transmission.


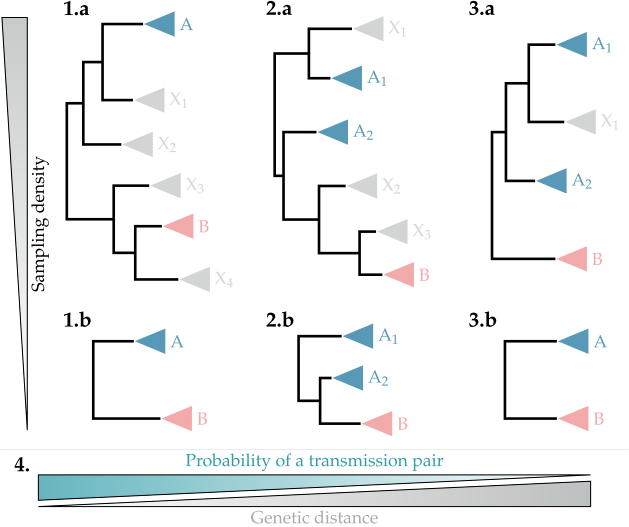


**Supplemental Figure S2.** Number of transmission clusters versus number of individuals for which a multiple infection would be predicted given a set of thresholds. The y-axis indicates the lower bound for the bootstrap support value of the clade. The maximum pairwise genetic distance is depicted on the x-axis. Each rectangle corresponds to one set of thresholds and shows the number of transmission clusters (upper left) versus number of co-infected individuals (lower right).


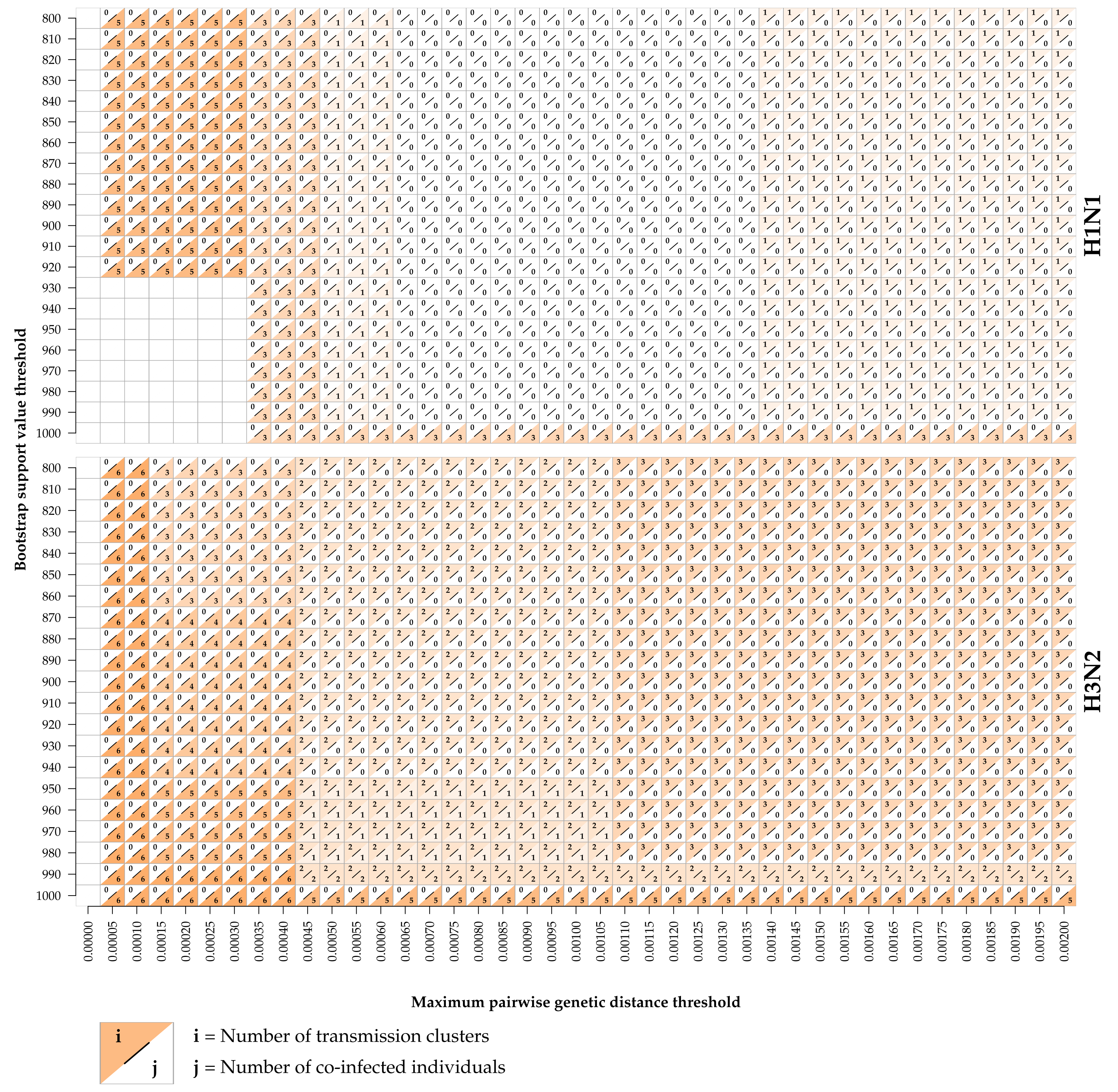


**Supplemental Figure S3.** Three assumptions considered in deductive reasoning for selecting the genetic distance threshold in decreasing order of strictness. On each subtree each gray shaded rectangle represent a set of sequences that would (under the corresponding genetic distance threshold) be identified as a transmission cluster. Sequences belonging to the same patient but not appearing within the same rectangle indicate a co-infection under the corresponding set of thresholds. The bootstrap support value threshold in all three premises is B=900.


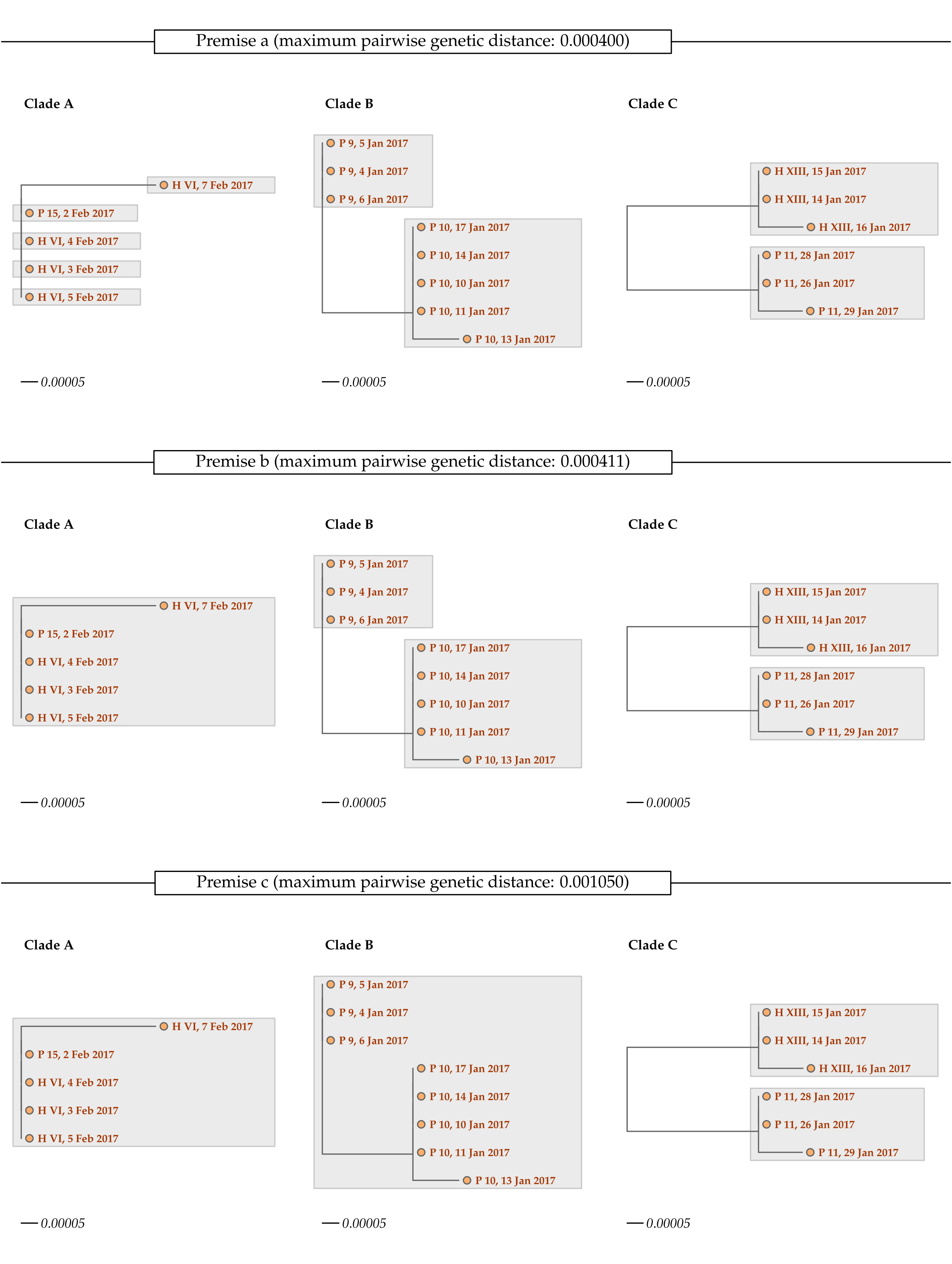


**Supplemental Table S3**. Adherence with the study protocol in 152 healthcare workers and 542 hospital inpatients participating in prospective surveillance for asymptomatic and symptomatic influenza infection during the 2015/16 and the 2016/17 influenza season, University Hospital Zurich.

| **Item** | **HCW**  **(N=152)** | **INPATIENTS**  **(N=542)** |
| --- | --- | --- |
| Number of swabs | 7,171 | 3,498 |
| 2015/16 season total | 3,503 | 1,480 |
| median (IQR) | 69 (31-85) | 5 (3-9.5) |
| 2016/17 season total | 3,668 | 2,018 |
| median (IQR) | 42 (21-57) | 5 (3-8) |
| Proportion of expected swabs %, median (IQR) | 73.1 (36.2-88.5) | 60.0 (43.8-71.4) |
| in hospital %, median (IQR) | n.a. | 81.3 (62.5-100) |
| Number of diaries | 7,490 | 3,526 |
| 2015/16 season total | 3,676 | 1,484 |
| median (IQR) | 72 (34-89) | 5 (3-9.5) |
| 2016/17 season total | 3,814 | 2,042 |
| median (IQR) | 43 (25-58) | 5 (3-8) |
| Proportion of expected diaries %, median (IQR) | 75.4 (41.5-92.7) | 60 (43.4-71.4) |
| in hospital %, median (IQR) | n.a. | 81.8 (65.5-100) |
| Abbreviations: HCW, healthcare worker; IQR, interquartile range; n.a. not applicable. | | |

**Supplemental Table S4.** Characteristics of healthcare workers and inpatients participating in prospective surveillance for asymptomatic and symptomatic influenza infection during the 2015/16 and the 2016/17 influenza season, University Hospital Zurich

| **Characteristic** | **HCW**  **(N=152)** | **INPATIENTS**  **(n=542)** |
| --- | --- | --- |
| 2015/16 season | 59 (38.8)^a^ | 192 (35.4) |
| 2016/17 season | 94 (61.8)^a^ | 350 (64.6) |
| Duration of study participation, median (range) |  |  |
| 2015/16 season | 96 (71-96) | 8 (3-86) |
| 2016/17 season | 66 (25-67) | 8 (3-44) |
| Age,years, median (range) | 31.0 (18.2-62.0) | 60.8 (18.2-95.6) |
| Female sex, n (%) | 124 (81.6) | 224 (41.3) |
| Profession, n (%) |  |  |
| Nurse | 100 (65.8) | n.a. |
| Physician | 20 (13.6) | n.a. |
| Physiotherapist | 27 (17.8) | n.a. |
| Corporate Hospitality | 5 (3.3) | n.a. |
| Influenza vaccine |  |  |
| Study season | 36 (23.7) | 173 (31.9) |
| Study season and prior season | 33 (21.7) | 155 (28.6) |
| Charlson comorbidity index, median (range) | 0 (0-1) | 2 (0-13) |
| Myocardial infarction | 0 (0) | 52 (9.6) |
| Congestive heart failure | 0 (0) | 100 (18.5) |
| Peripheral vascular disease | 0 (0) | 37 (6.8) |
| Chronic pulmonary disease | 4 (2.6) | 144 (26.6) |
| Connective tissue disease | 0 (0) | 21 (3.9) |
| Diabetes without complications | 1 (0.7) | 79 (14.6) |
| Peptic ulcer disease | 1 (0.7) | 3 (0.6) |
| Chronic disease of the liver or cirrhosis | 0 (0) | 5 (0.9) |
| Hemiplegia | 0 (0) | 1 (0.2) |
| Moderate or severe kidney disease | 0 (0) | 43 (7.9) |
| Diabetes with chronic complications | 0 (0) | 22 (4.1) |
| Tumors | 0 (0) | 53 (9.8) |
| Leukemia | 0 (0) | 30 (5.5) |
| Lymphoma | 0 (0) | 27 (5.0) |
| Moderate or severe liver disease | 0 (0) | 44 (8.1) |
| Malignant tumor, metastasis | 0 (0) | 53 (9.8) |
| AIDS | 0 (0) | 6 (1.1) |
| Dementia | 0 (0) | 1 (0.2) |
| Hausehold crowding index, median (range) | 1.3 (0.5-3) | n.a. |
| Kids in household | 34 (22.4) | n.a. |
| Data are n(%) unless indicated otherwise. ^a^1 HCW participated in both seasons.Abbreviations: n.a., not applicable. | | |

**Supplemental Figure S4.** Overview of study workflow and results


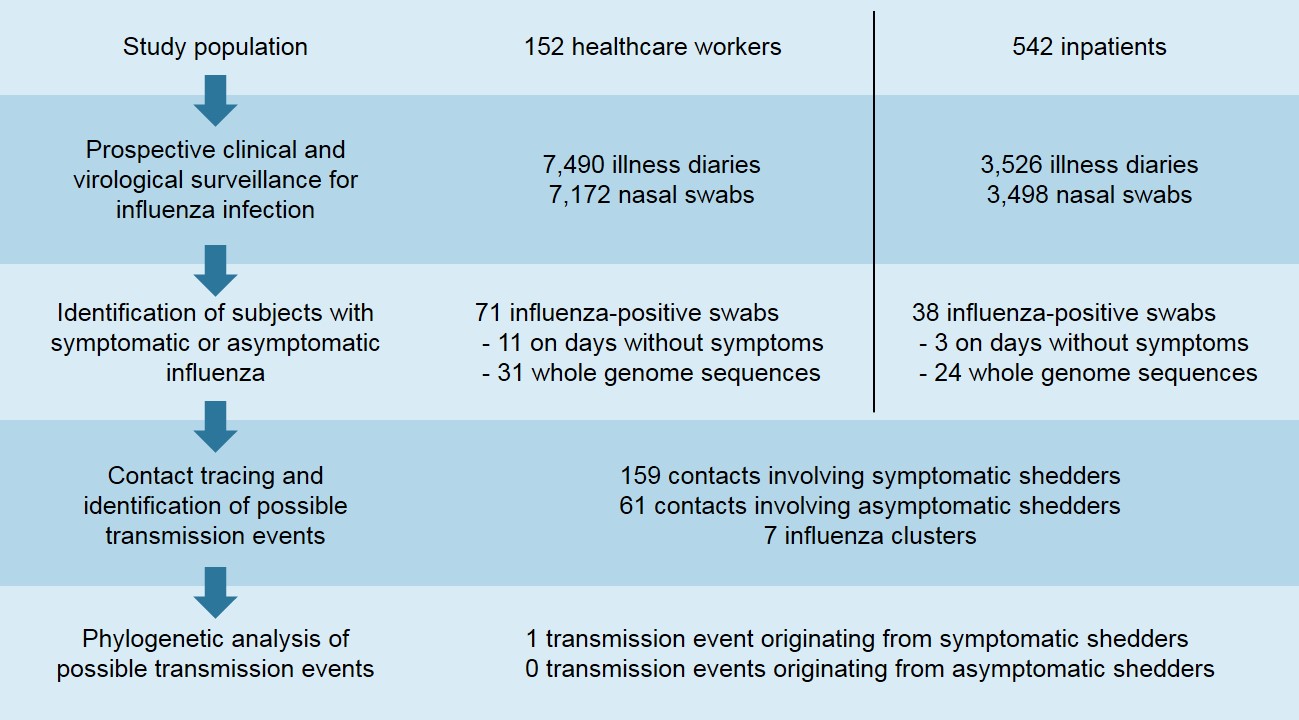


**Supplemental Figure S5.** Phylogenetic trees of near full-length genome sequences of influenza virus collected at the University Hospital Zurich in the 2015/16 and 2016/17 influenza seasons


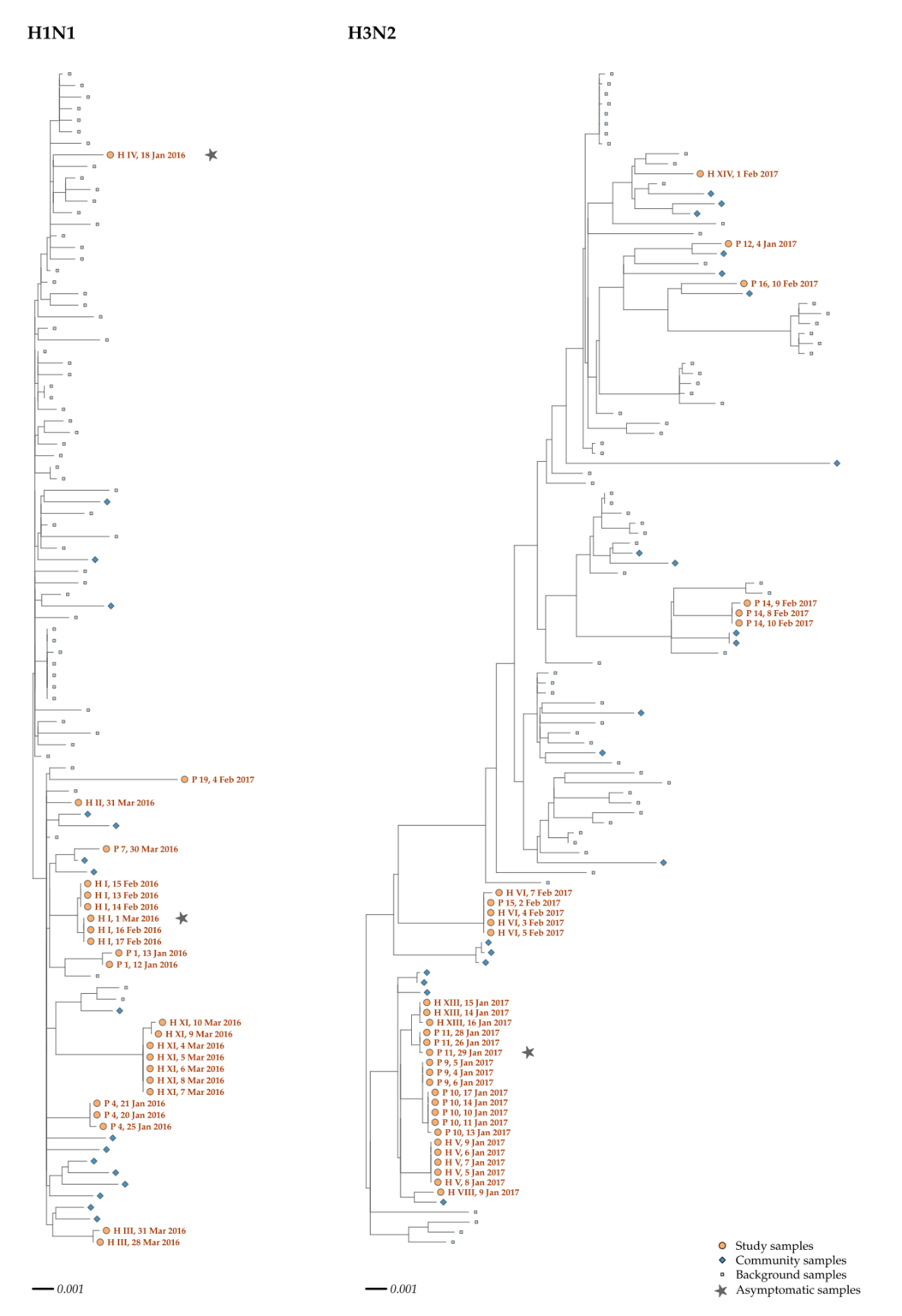

Supplement: Supplementary file 1 [file S0899823X21001124sup001.docx]
